# Network meta-analysis of the efficacy of first-line chemotherapy regimens in patients with advanced colorectal cancer

**DOI:** 10.18632/oncotarget.22177

**Published:** 2017-10-31

**Authors:** Dong-Mei Wu, Yong-Jian Wang, Shao-Hua Fan, Juan Zhuang, Zi-Feng Zhang, Qun Shan, Xin-Rui Han, Xin Wen, Meng-Qiu Li, Bin Hu, Chun-Hui Sun, Ya-Xing Bao, Hai-Juan Xiao, Lin Yang, Jun Lu, Yuan-Lin Zheng

**Affiliations:** ^1^ Key Laboratory for Biotechnology on Medicinal Plants of Jiangsu Province, School of Life Science, Jiangsu Normal University, Xuzhou 221116, P.R. China; ^2^ School of Environment Science and Spatial Informatics, China University of Mining and Technology, Xuzhou 221008, P.R. China; ^3^ Jiangsu Key Laboratory for Eco-Agricultural Biotechnology around Hongze Lake, School of Life Sciences, Huaiyin Normal University, Huaian 223300, P.R. China; ^4^ Department of Orthopaedics, The Affiliated Municipal Hospital of Xuzhou Medical University, Xuzhou 221009, P.R. China; ^5^ Department of Oncology, Hospital Affiliated to Shaanxi University of Chinese Medicine, Xianyang 712000, P.R. China; ^6^ Department of Hepatobiliary Surgery, Xianyang Central Hospital, Xianyang 712000, P.R. China

**Keywords:** advanced colorectal cancer, chemotherapy, efficacy, randomized controlled trial, bayesian network model

## Abstract

This network meta-analysis compared the short-term and long-term efficacies of first-line chemotherapy regimens in patients with advanced colorectal cancer (CRC). The 10 regimens included folinic acid + 5-fluorouracil + oxaliplatin (FOLFOX), folinic acid + 5-fluorouracil + irinotecan (FOLFIRI), folinic acid + 5-fluorouracil + gemcitabine (FFG), folinic acid + 5-fluorouracil + trimetrexate (FFT), folinic acid + 5-fluorouracil (FF), irinotecan + oxaliplatin (IROX), raltitrexed + oxaliplatin (TOMOX), folinic acid + tegafur-uracil (FTU), raltitrexed, and capecitabine. Electronic searches were performed in the Cochrane Library, PubMed and Embase databases from inception to June 2017. Network meta-analysis combined direct and indirect evidence to obtain odds ratios (ORs) and surface under the cumulative ranking curves (SUCRA) of different chemotherapy regimens for advanced CRC. Fourteen randomized controlled trails (RCTs) covering 4,383 patients with advanced CRC were included. The results revealed that FOLFOX, FOLFIRI, IROX, and TOMOX all showed higher overall response rates (ORRs) than FF or raltitrexed. Compared with raltitrexed, the aforementioned four regimens also had higher 1-year progression-free survival (PFS) rates. In addition, FOLFOX and FOLFIRI exhibited higher disease control rates (DCRs) and 1-year PFS rates than FF or raltitrexed. Cluster analysis revealed that FOLFOX, FOLFIRI, and TOMOX had better short-term and long-term efficacies. These findings suggest FOLFOX, FOLFIRI, and TOMOX are superior to other regimens for advanced CRC. These three regimens are therefore recommended for clinical treatment of advanced CRC.

## INTRODUCTION

Colorectal cancer (CRC) is the most commonly diagnosed cancer and is the fourth leading cause of cancer-related deaths all over the world [1, 2]. According to the World Health Organization (WHO) estimates for 2030, CRC new cases will increase by 77% annually and CRC-related deaths will increase by 80% [3]. CRC results from benign neoplasms, such as tubular adenomas and serrated polyps, which evolve into CRC over many years and undergo malignant transformation [4]. CRC is preventable with early diagnosis, and currently, medical imaging is an important modality for screening, staging, and surveillance of CRC [5]. The most often treatments of CRC can be surgical resection, adjuvant chemotherapy, and drug therapy [1]. Because of the complex structure of the pelvis, the treatment of CRC is not completed by the surgical operation, so drug-combination chemotherapy has also become a common treatment of CRC [6].

Drug chemotherapy can inhibit the proliferation of tumor cells when the chemical compounds are directed at the signal pathways in tumor cells [7]. Although 5-fluorourcacil (5-FU) is widely used in the treatment of CRC, drug resistance limits its clinical application [8], which means that 5-FU alone has low efficacy in the treatment of CRC [9]. Studies indicate that regimens containing cytotoxic drugs such as raltitrexed, oxaliplatin, and irinotecan could improve patient outcomes [10, 11]. Consequently, researchers have developed important insight into combining various compounds to improve therapeutic efficacy and reduce the side effects of drugs [12]. A study demonstrated that when associated with folinic acid, 5-FU enhances the clinical response, and when combined with irinotecan or oxaliplatin, 5-FU increases survival rates in CRC patients [13]. When 5-FU is combined with either agent, response rates (RRs) increase from 15%–25% to 40%–50%, and overall survival (OS) is prolonged to more than 20 months. When three active agents such as 5-FU, irinotecan, and oxaliplatin are combined in one regimen, the combination shows only partly overlapping toxicity profiles, which means that a triple combination might be feasible [14]. However, the combination of various drugs has not achieved significant improvement [15], suggesting that the most effective therapeutic regimen for the treatment and management of CRC remains to be determined.

A network meta-analysis can summarize all included research results, and then an integrated analysis of intervention experiments can be performed to establish a net-like relation to analyze efficiencies of multiple interventions in the disease and to screen for valuable results [16]. It is well known that the targeted drug is really a feasible and efficient assisted strategy and chemotherapeutic drugs exhibit many varieties and some relevant researches increasingly updated [17], Wondering whether there exist chances of the combination of various chemotherapeutic drugs and with the aim of producing more reliable guidance for the drug regimens for advanced CRC, we thus perform a network meta-analysis to compare the short-term and long-term efficacies of different first-line chemotherapy regimens in the treatment of advanced CRC.

## RESULTS

### Baseline characteristics of included studies

**Figure 1:**
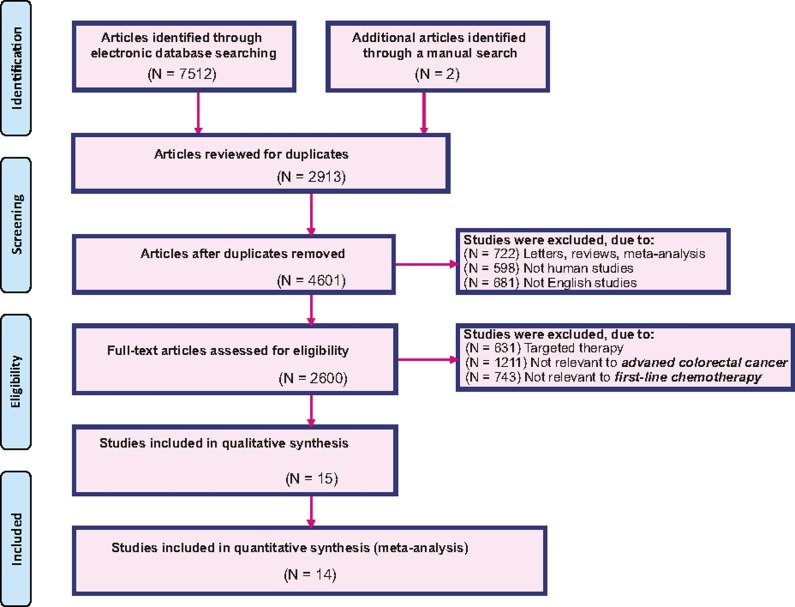
Flowchart of the literature search and screening results

Through electronic and manual searches, 7,514 articles were found. After the initial screening, we excluded 2,913 duplicated articles, 722 letters or summaries, 598 articles on non-human studies, and 681 non-English articles. For the remaining 2,600 articles, after detailed assessment of the full text, we excluded 631 articles on targeted therapy, 1,211 articles that were irrelevant with the advanced CRC, 743 articles that were irrelevant with chemotherapy, and 1 article with no data or insufficient data. Eventually, 14 eligible randomized control trials (RCTs) which were published between 1996 and 2015 were included for this network meta-analysis (Figure 1) [18–31]. All these studies objects were from European and American populations and the 14 studies were part of two-arm trials. The baseline characteristics of included studies are shown in Table 1, and the PEDro scale for literature quality assessment is displayed in Supplementary Figure 1.

**Table 1:** The baseline characteristics for included studies

First author	Year	Country	Phase	Interventions		Total	Sample size		Gender (M/F)		Age (years)	
				T1	T2		T1	T2	T1	T2	T1	T2
Kroep JR	2015	The Netherlands	Phase III	H	J	67	34	33	19/15	17/16	77 (66–88)	76 (66–88)
Madajewicz S	2012	America	Phase II	A	C	48	24	24	9/15	10/14	64 (32–81)	63 (28–79)
Gravalos C	2012	Spain	Phase II	A	G	183	91	92	48/43	56/36	61 (35–82)	65 (36–78)
Fischer von WL	2011	Germany	Phase III	B	F	479	238	241	158/80	177/64	63 (32–79)	63 (21–79)
Hospers GA	2006	The Netherlands	Phase III	A	E	302	151	151	100/51	88/63	62 (41–80)	62 (28–84)
Kalofonos HP	2005	Greece	Phase II	A	B	295	148	147	92/56	90/57	65 (28–78)	66 (28–78)
Colucci G	2005	Italy	Phase III	A	B	360	182	178	109/73	93/85	62 (31–75)	62 (32–75)
Tournigand C	2004	France	Phase III	A	B	220	111	109	80/31	62/47	65 (40–75)	61 (29–75)
Scheithauer W	2002	Austria	Phase II	F	I	92	46	46	27/19	22/24	65 (38–75)	68 (31–75)
Blanke CD	2002	Portland	Phase III	D	E	382	191	191	61/39	64/36	66 (22–86)	63 (25–90)
Twelves C	2001	England	Phase III	E	J	602	301	301	173/128	172/129	63.5 (36–86)	62(29–84)
de Gramont A	2000	France	Phase III	A	E	420	210	210	127/83	122/88	63 (20–76)	63(22–76)
Cocconi G	1998	Italy	Phase II	E	I	495	248	247	164/84	152/95	62 (36–83)	60 (23–79)
Cunningham D	1996	England	Phase III	E	I	439	216	223	127/89	133/90	61 (27–80)	61 (27–82)

Note: T = treatment; M = male; F = female; A = FOLFOX (folinic acid + 5-fluorouracil + oxaliplatin); B = FOLFIRI (folinic acid + 5-fluorouracil+irinotecan); C = FFG (folinic acid + 5-fluorouracil + gemcitabine); D = FFT (folinic acid + 5-fluorouracil+trimetrexate); E = FF (folinic acid + 5-fluorouracil); F = IROX (irinotecan + oxaliplatin); G = TOMOX (raltitrexed + oxaliplatin); H = FTU (folinic acid + tegafur-uracil); I = raltitrexed; J = capecitabine.

### Pairwise meta-analysis of ORR, DCR, 1-year OS rate, 2-year OS rate, 1-year PFS rate, and 2-year PFS rate for patients with advanced CRC

The direct paired comparisons were performed for the short-term and long-term efficacies of 10 first-line chemotherapy regimens in the treatment of advanced CRC, and the results indicated that for ORR, DCR, and 1-year PFS rate, the efficacy of FF was poor compared with FOLFOX (OR = 2.97, 95% CI = 2.14–4.14; OR = 1.91, 95% CI = 1.34–2.71; OR = 2.34, 95% CI = 1.61–3.41, respectively) (Supplementary Table 1). However, in terms of 1-year OS rate, 2-year OS rate, and 2-year PFS rate, there was no significant difference in the results. Overall, the efficacy of FOLFOX chemotherapy was much better, while FF chemotherapy regimen was poor for patients with advanced CRC.

### Evidence network of ORR, DCR, 1-year OS rate, 2-year OS rate, 3-year OS rate, 1-year PFS rate, and 2-year PFS rate for patients with advanced CRC

**Figure 2:**
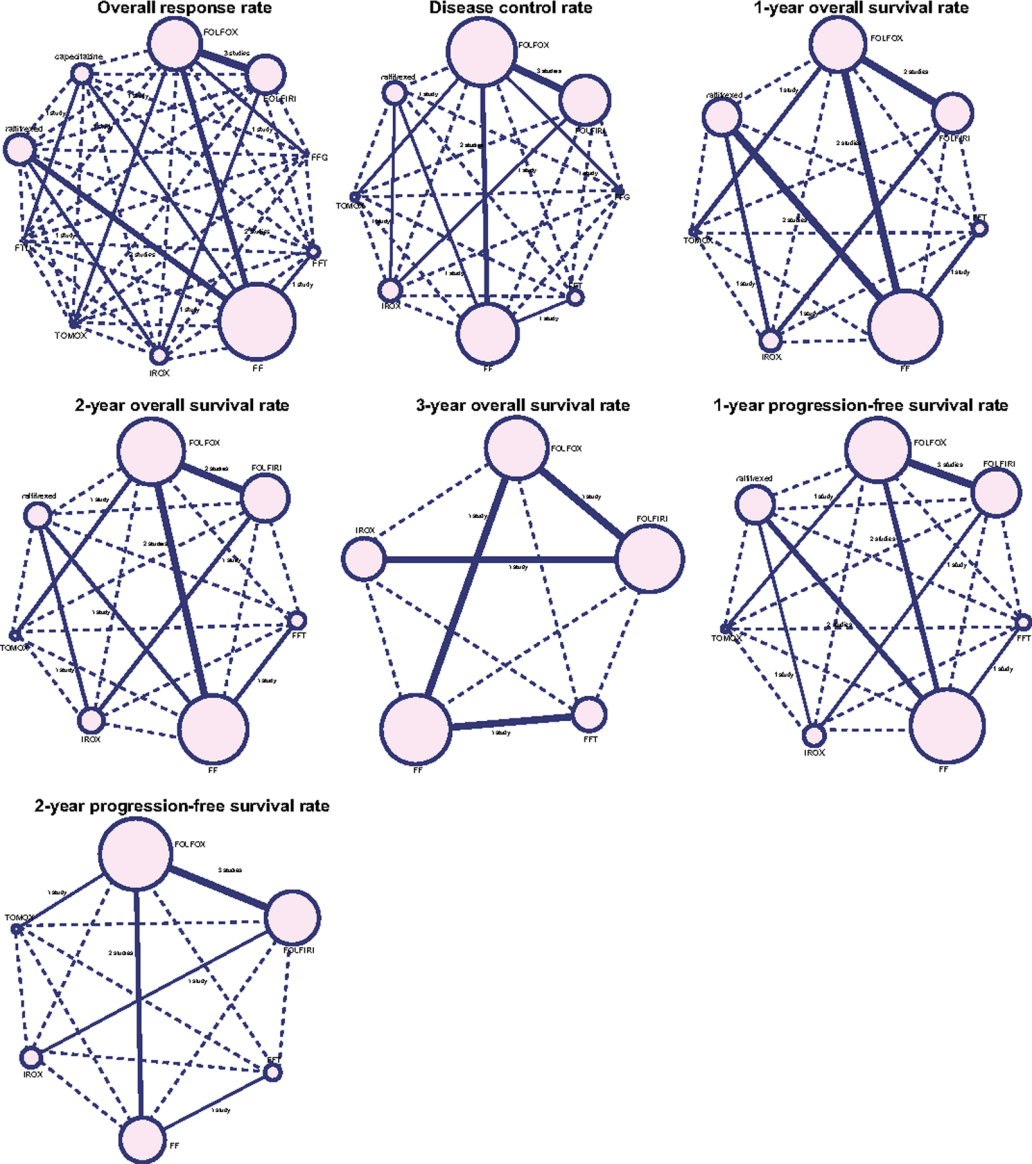
Network diagrams of ORR, DCR, 1-year OS rate, 2-year OS rate, 3-year OS rate, 1-year PFS rate, and 2-year PFS rate Note: ORR, overall response rate; DCR, disease control rate; OS, overall survival; PFS, and progression-free survival (FOLFOX = folinic acid + 5-fluorouracil + oxaliplatin, FOLFIRI = folinic acid + 5-fluorouracil + irinotecan, FFG = folinic acid + 5-fluorouracil + gemcitabine, FFT = folinic acid + 5-fluorouracil + trimetrexate, FF = folinic acid + 5-fluorouracil, IROX = irinotecan + oxaliplatin, TOMOX = raltitrexed + oxaliplatin, and FTU = folinic acid + tegafur-uracil).

This study included 10 first-line chemotherapy regimens: FOLFOX, FOLFIRI, FFG, FFT, FF, IROX, TOMOX, FTU, raltitrexed, and capecitabine. More advanced CRC patients were treated with FOLFOX, FOLFIRI, and FF first-line chemotherapy regimens. In addition, more studies compared FOLFOX with FOLFIRI, FOLFOX with FF, and FF with raltitrexed (Figure 2).

### Inconsistency test of ORR, DCR, 1-year OS rate, 2-year OS rate, 3-year OS rate, 1-year PFS rate, and 2-year PFS rate for patients with advanced CRC

The node-splitting method was used for the inconsistency test of ORR, DCR, 1-year OS rate, 2-year OS rate, and 1-year PFS rate, and the results demonstrated that all direct evidence and indirect evidence were consistent, suggesting the consistency model should be adopted (all *P* > 0.05) (Table 2).

**Table 2:** OR values and *P* values of direct and indirect pairwise comparisons among five treatment modalities under five endpoint outcomes

Pairwise comparisons	Direct OR values					Indirect OR values					*P* values				
	B vs. A	E vs. A	F vs. B	I vs. E	I vs. F	B vs. A	E vs. A	F vs. B	I vs. E	I vs. F	B vs. A	E vs. A	F vs. B	I vs. E	I vs. F
**ORR**	1.00	0.34	1.00	1.10	0.31	1.20	0.27	1.20	0.93	0.37	0.81	0.78	0.83	0.79	0.84
**DCR**	1.10	0.52	0.50	0.97	0.36	2.80	0.21	1.20	0.39	0.93	0.20	0.22	0.24	0.21	0.21
**1-year OS rate**	0.97	0.81	0.72	0.87	0.94	1.10	0.72	0.81	0.79	1.00	0.88	0.87	0.85	0.85	0.89
**2-year OS rate**	1.20	0.83	0.68	0.81	0.77	1.30	0.72	0.77	0.74	0.87	0.86	0.83	0.82	0.87	0.85
**1-year PFS rate**	0.82	0.43	0.77	0.86	0.13	3.90	0.09	3.50	0.18	0.55	0.13	0.14	0.15	0.14	0.16

Notes: OR = odds ratio; ORR = overall response rate; DCR = disease control rate; OS = overall survival; PFS = progression-free survival; A = FOLFOX (folinic acid + 5-fluorouracil + oxaliplatin); B = FOLFIRI (folinic acid + 5-fluorouracil + irinotecan); E = FF (folinic acid + 5-fluorouracil); F = IROX (irinotecan + oxaliplatin); I = raltitrexed; J = capecitabine.

### Network meta-analysis of ORR, DCR, 1-year OS rate, 2-year OS rate, 3-year OS rate, 1-year PFS rate, and 2-year PFS rate for patients with advanced CRC

As shown in Supplementary Table 2, network meta-analysis results revealed that FOLFOX, FOLFIRI, IROX, and TOMOX had better efficacies for patients with advanced CRC. The ORRs of these four chemotherapy regimens were higher than the ORR of FF (OR = 2.95, 95% CI = 1.95–4.65; OR = 3.11, 95% CI = 1.81–5.32; OR = 3.14, 95% CI = 1.61–6.33; and OR = 4.36, 95% CI = 1.94–10.49, respectively) and raltitrexed (OR = 2.78, 95% CI = 1.58–5.02; OR = 2.93, 95% CI = 1.54–5.47; OR = 2.92, 95% CI = 1.47–5.89; and OR = 4.13, 95% CI = 1.66–10.22, respectively). Compared with raltitrexed, 1-year PFS rates of FOLFOX, FOLFIRI, IROX, and TOMOX were higher (OR = 3.66, 95% CI = 1.62–9.74; OR = 3.26, 95% CI = 1.34–10.09; OR = 3.02, 95% CI = 1.24–11.41; and OR = 3.76, 95% CI = 1.04–16.72, respectively). In addition, compared with raltitrexed, the DCR was higher in FOLFOX and FOLFIRI (OR = 2.11, 95% CI = 1.02–5.18 and OR = 2.59, 95% CI = 1.18–6.60, respectively). Compared with FF, 1-year PFS rate was higher in FOLFOX and FOLFIRI (OR = 2.59, 95% CI = 1.36–5.36 and OR = 2.36, 95% CI = 1.08–5.80, respectively). It can be concluded that the efficacy of FOLFOX, FOLFIRI, IROX, and TOMOX were much better for patients with advanced CRC.

### SUCRA of ORR, DCR, 1-year OS rate, 2-year OS rate, 3-year OS rate, 1-year PFS rate, and 2-year PFS rate for patients with advanced CRC

As shown in Table 3, the highest SUCRA values for ORR, DCR, 1-year OS rate, and 2-year OS rate (ORR: 91.8%, DCR: 88.3%, 1-year OS rate: 78.1%, and 2-year OS rate: 90.3%, respectively) were found in TOMOX. For 1-year PFS rate and 2-year PFS rate, FOLFOX had the highest SUCRA values (1-year PFS rate: 84.4% and 2-year PFS rate: 76.0%). For 3-year OS rate, FOLFIRI achieved the highest SUCRA value (81.0%). Overall, FOLFOX, FOLFIRI, and TOMOX regimens were more effective in the treatment of patients with advanced CRC.

**Table 3:** SUCRA values of ten treatment modalities under seven endpoint outcomes

Treatments	SUCRA values (%)						
	ORR	DCR	1-year OS rate	2-year OS rate	3-year OS rate	1-year PFS rate	2-year PFS rate
**A**	75.5	74.9	74.3	64.4	75.2	**84.4**	**76.0**
**B**	78.6	85.9	69.9	79.6	**81.0**	75.0	51.8
**C**	30.9	52.6	NR	NR	NR	NR	NR
**D**	41.1	30.1	65.9	47.4	41.0	30.3	67.8
**E**	21.9	34.8	47.6	45.9	36.0	37.7	56.2
**F**	77.6	55.6	34.4	44.4	67.0	70.0	33.3
**G**	**91.8**	**88.3**	**78.1**	**90.3**	NR	80.9	65.3
**H**	56.5	NR	NR	NR	NR	NR	NR
**I**	26.5	27.3	30.9	27.7	NR	22.1	NR
**J**	48.1	NR	NR	NR	NR	NR	NR

Notes: SUCRA = surface under the cumulative ranking curves. ORR = overall response rate; DCR = disease control rate; OS = overall survival; PFS = progression-free survival; A = FOLFOX (folinic acid + 5-fluorouracil + oxaliplatin); B = FOLFIRI (folinic acid + 5-fluorouracil + irinotecan); C = FFG (folinic acid + 5-fluorouracil + gemcitabine); D = FFT (folinic acid + 5-fluorouracil + trimetrexate); E = FF (folinic acid + 5-fluorouracil); F = IROX (irinotecan + oxaliplatin); G = TOMOX (raltitrexed + oxaliplatin); H = FTU (folinic acid + tegafur-uracil); I = raltitrexed; J = capecitabine.

### Cluster analysis of ORR, DCR, 1-year OS rate, 2-year OS rate, 3-year OS rate, 1-year PFS rate, and 2-year PFS rate for patients with advanced CRC

**Figure 3:**
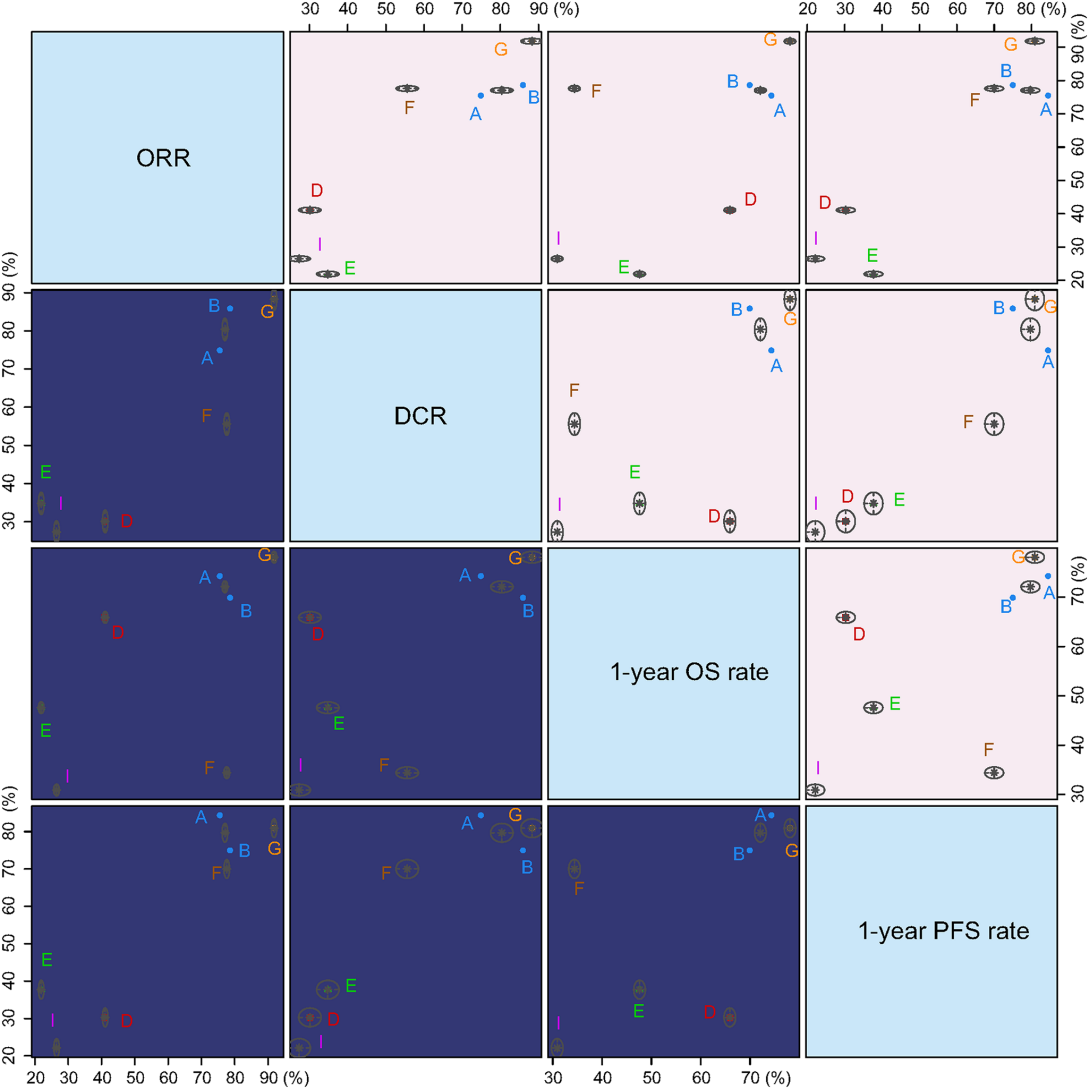
Cluster analysis diagrams of ORR, DCR, 1-year OS rate, and 1-year PFS rate Note: ORR, overall response rate; DCR, disease control rate; OS, overall survival; and PFS, progression-free survival. (**A**) FOLFOX (folinic acid + 5-fluorouracil + oxaliplatin), (**B**) FOLFIRI (folinic acid + 5-fluorouracil + irinotecan), (**C**) FFG (folinic acid + 5-fluorouracil + gemcitabine), (**D**) FFT (folinic acid + 5-fluorouracil + trimetrexate), (**E**) FF (folinic acid + 5-fluorouracil), (**F**) IROX (irinotecan + oxaliplatin), (**G**) TOMOX (raltitrexed + oxaliplatin), (**H**) FTU (folinic acid + tegafur-uracil), (**I**) raltitrexed, and (**J**) capecitabine.

Cluster analysis was performed for SUCRA values of ORR, DCR, 1-year OS rate, and 1-year PFS rate. The results illustrated that compared with other chemotherapy regimens, FOLFOX, FOLFIRI, and TOMOX had better short-term and long-term efficacies. The FOLFOX and FOLFIRI values were clustered, and the efficacy of FF and raltitrexed was poor for patients with advanced CRC (Figure 3).

## DISCUSSION

This study conducted a network meta-analysis of 10 first-line chemotherapy regimens to compare the short-term and long-term efficacies in patients suffering from advanced CRC. The analysis results demonstrated that FOLFOX, FOLFIRI, and TOMOX achieved better outcomes in the treatment of advanced CRC when compared with FFG, FF, and IROX.

FOLFOX, FOLFIRI, and TOMOX had better short-term and long- term efficacies. FOLFOX, FOLFIRI, and TOMOX had higher ORRs and 1-year PFS rates. Hideo *et al.* [32] demonstrated that the FOLFOX4 regimen shows good efficacy with an acceptable overall toxicity profile in a Japanese population. Iwamoto [33] reported that the FOLFIRI regimen as first-line treatment shows better response rate efficacy (41%), time to progression (6.7 months), and median survival time (17.4 months). Furthermore, FOLFIRI had better DCR and 2-year OS rate efficacies. Ludwig *et al.* [21] demonstrated that high-dose folinic acid/5-fluorouracil plus irinotecan shows superior activity, which appeared to have comparable clinical activity for ORR, PFS, and OS, which is consistent with our results. In addition, TOMOX had the highest SUCRA values for ORR, DCR, 1-year OS rate, and 2-year OS rate. TOMOX had a higher ORR. Martoni *et al.* [34] reported that the combination of raltitrexed and oxaliplatin is active in advanced CRC, which is consistent with our findings. Moreover, Sandro *et al.* [35] showed that TOMOX has better RR (45%) and is active as a first-line chemotherapy regimen for advanced CRC. The chemotherapeutic mechanisms of oxaliplatin and irinotecan function by disrupting related pathways of epidermal growth factor receptor (EGFR) and vascular endothelial growth factor (VEGF) [36], which inhibits further proliferation and metastasis of tumors to achieve a meaningful efficacy. Oxaliplatin is a novel platinum derivative that suppresses DNA replication *via* the formation of DNA adducts, and it has activity in advanced tumor treatment both in combination and in monotherapy arms [37]. Raltitrexed is a specific inhibitor of thymidylate synthase (TS). Raltitrexed is polyglutamated *via* folylpolyglutamate synthase and enters cells through the reduced-folate carrier, which enhances intracellular retention and results in prolonged TS inhibition, DNA fragmentation, and cell death [20]. To some extent, the results of this study are reasonable and can provide support for selection of effective chemotherapy regimens in the treatment of advanced CRC. Additionally, FF and raltitrexed regimens showed relatively poor efficacy in the treatment of advanced CRC. FF and raltitrexed had lower ORRs and 1-year PFS rates. Twelves *et al.* [28] demonstrate that the ORR is lower for patients treated with FF than for patients receiving capecitabine (17.9% vs. 26.6%). When compared with FF, raltitrexed causes more thrombocytopenia and elevated liver transaminases [38].

The Bayesian network model is applied for an inconsistency test of direct and indirect evidence by using the node-splitting method. With this method, we can avoid the shortcomings of the traditional meta-analysis, which can only directly compare two different interventions to conduct direct and indirect comparisons of all the results, and thus achieve more complete analysis results [39]. Moreover, the test results showed all the direct and indirect evidence are consistent, suggesting that the results of this study are reliable. Still, due to the limited relative literatures, some included studies lacking sufficient comparisons may have a certain impact on the result. In addition, the collected results from the included studies were uneven and the number of studies on some drugs was small. Such a situation causes inconsistency between pairwise meta-analysis conclusions and network meta-analysis conclusions. Because of the limitations in our study, we must collect more data from diagnostic studies to improve the results.

This study found that multi-drug regimens are superior to single-drug regimens for advanced CRC. Furthermore, FOLFOX, FOLFIRI, and TOMOX are much more beneficial for the treatment of advanced CRC.

## MATERIALS AND METHODS

### Literature search

This systematic review was performed according to Preferred Reporting Items for Systematic Reviews and Meta-Analyses (PRISMA) guidelines [40]. Electronic searches were performed in the Cochrane Library, PubMed and Embase databases from the inception to June 2017. We also searched for relevant studies that were missed in the initial electronic search by conducting a manual search of cross-references. The manual search was conducted using keywords combined with free words, mainly including chemotherapy, pharmacotherapy, oxaliplatin, fluorouracil, irinotecan, capecitabine, colorectal cancer etc.

### Inclusion and exclusion criteria

The inclusion criteria were: (1) study design: randomized controlled trail (RCT); (2) chemotherapy regimens: folinic acid + 5-FU + oxaliplatin (FOLFOX), folinic acid + 5-FU + irinotecan (FOLFIRI), folinic acid + 5-FU + gemcitabine (FFG), folinic acid + 5-FU + trimetrexate (FFT), folinic acid + 5-FU (FF), irinotecan + oxaliplatin (IROX), raltitrexed + oxaliplatin (TOMOX), folinic acid + tegafur-uracil (FTU), raltitrexed, and capecitabine; (3) study subjects: patients with advanced CRC and at least one measurable lesion according to the Response Evaluation Criteria in Solid Tumors (RECIST) Version 1.0 [41]; (4) end outcomes: overall response rate (ORR), disease control rate (DCR), 1-year OS rate, 2-year OS rate, 3-year OS rate, 1-year progression-free survival (PFS) rate, or 2-year progression-free survival rate [42]. The exclusion criteria were: (1) studies without sufficient data, such as non-match researches; (2) non-RCTs; (3) duplicated publications; (4) conference reports, system assessments or abstracts; (5) studies investigating second-line chemotherapy regimens in the treatment of advanced CRC; (6) non-English literature; and (7) non-human studies.

### Data extraction and quality evaluation

With uniform data collection sheets, two reviewers independently extracted information from the selected studies. Any disputes regarding the extraction of data were resolved by agreement among several investigators. Literature quality was assessed by over two reviewers in accordance with the Physiotherapy Evidence Database (PEDro) scale, which has 11 total points (≥4 points, high quality; <4 points, low quality) [43].

### Statistical analysis

We conducted pair-wise meta-analyses of direct evidence by using the fixed-effects model supplemented with R Version 3.2.1 software and the meta-analysis package. The pooled estimates of odds ratios (ORs) and 95% confidence intervals (CIs) of seven endpoint outcomes were shown. The Chi-squared test and the I-squared test were used to test heterogeneity among the studies [44]. R 3.2.1 was applied with the network package to a draw net-like relation graph, in which each node refers to one intervention, node size refers to sample size, and line thickness between nodes refers to the quantity of enrolled studies. A random-effects network meta-analysis was performed by application of the GEMTC package. Lu and Ades [45] described random-effects network meta-analysis models, with the relative effects (e.g., log-odds ratio) fitting a generalized linear model (GLM) under the Bayesian framework by linking to JAGS, OpenBUGS, or WinBUGS. We used the node-splitting method to estimate consistency between the direct evidence and indirect evidence. Based on the results, a consistency or an inconsistency model was selected. When the results of node-splitting methods were *P* > 0.05, a consistency model was selected for the analysis [46]. To assist with the interpretation of ORs, we calculated the probability of each intervention that was the most effective treatment method based on a Bayesian approach using probability values summarized as surface under the cumulative ranking curves (SUCRA). That is, the larger the SUCRA value, the better the rank of the intervention [47, 48]. Cluster analyses were used to group the treatments on the basis of their similarity to primary and secondary outcomes [47]. All analyses were performed with R 3.2.1 software.

## SUPPLEMENTARY MATERIALS FIGURE AND TABLES




