# Supplementary material for: Network meta-analysis of the efficacy of first-line chemotherapy regimens in patients with advanced colorectal cancer
Source: Oncotarget. 2017 Oct 31;8(59):100668–77. doi: 10.18632/oncotarget.22177 (PMC5725053; doi:10.18632/oncotarget.22177)
Supplement: Supplementary file 1 [file oncotarget-08-100668-s001.pdf]

Network meta-analysis of the efficacy of first-line chemotherapy regimens in patients with advanced colorectal cancer

SUPPLEMENTARY MATERIALS

Supplementary Table 1: Estimated OR and 95% CI from pairwise meta-analysis

Included studies	Comparisons	Efficacy events		Pairwise meta-analysis		
		Treatment1	Treatment2	OR (95% CI)	I^2	P_h
**ORR (%)**						
2 studies	A vs. E	156/361	84/361	**2.97 (2.14–4.14)**	44.2%	0.181
3 studies	A vs. B	168/431	165/420	0.99 (0.75–1.31)	0%	0.849
2 studies	E vs. I	81/464	89/470	0.91 (0.65–1.26)	0%	0.669
**DCR (%)**						
2 studies	A vs. E	223/361	181/361	**1.91 (1.34–2.71)**	0%	0.430
3 studies	A vs. B	240/431	245/420	0.90 (0.67–1.19)	0%	0.807
**1-year OS rate (%)**						
2 studies	A vs. E	227/361	209/361	1.24 (0.91–1.70)	0%	0.447
2 studies	A vs. B	208/320	199/311	1.05 (0.75–1.46)	5.5%	0.304
2 studies	E vs. I	220/464	206/470	1.15 (0.89–1.49)	0%	0.625
**2-year OS rate (%)**						
2 studies	A vs. E	100/361	87/361	1.21 (0.86–1.69)	0%	0.474
2 studies	A vs. B	93/320	100/311	0.87 (0.62–1.22)	0%	0.953
**1-year PFS rate (%)**						
2 studies	A vs. E	102/361	52/361	**2.34 (1.61–3.41)**	0%	0.392
3 studies	A vs. B	105/431	89/420	1.20 (0.87–1.66)	0%	0.659
2 studies	E vs. I	36/464	32/470	1.15 (0.60–2.42)	45.0%	0.178
**2-year PFS rate (%)**						
2 studies	A vs. E	22/361	16/361	1.40 (0.72–2.74)	0%	0.336
3 studies	A vs. B	34/431	24/420	1.38 (0.79–2.41)	1.6%	0.362

Notes: OR = odds ratio; CI = confidence intervals; NA = not available; ORR = overall response rate; DCR = disease control rate; OS = overall survival; PFS = progression-free survival; A = FOLFOX (folinic acid + 5-fluorouracil + oxaliplatin); B = FOLFIRI (folinic acid + 5-fluorouracil + irinotecan); E = FF (folinic acid + 5-fluorouracil); I = raltitrexed.

Supplementary Table 2: OR (95% CI) of ten treatment modalities of seven endpoints. See Supplementary_Table_2

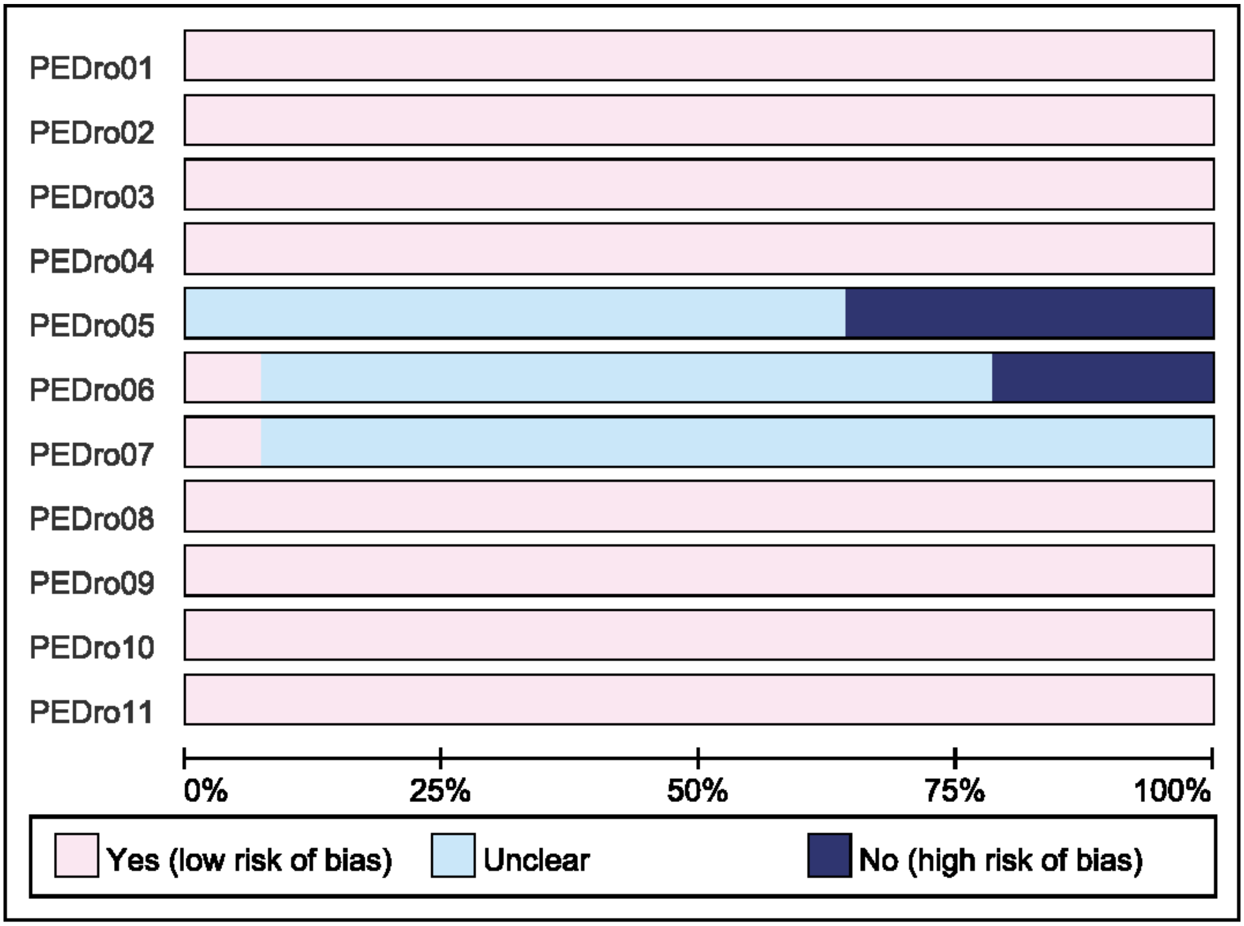

1. Eligibility criteria were specifcd
2. Subjects were randomly allocated to groups (in a crossover study, subjects were randomly allocated an order in which treatments were received)
3. Allocation was concealed
4. The groups were similar at baseline regarding the most important prognostic indicators
5. There was blinding of all subjects
6. There was blinding of all therapists who administered the therapy
7. There was blinding of all assessors who measured at least one key outcome
8. Measurements of at least one key outcome were obtained from more than 85% of the subjects initially allocated to groups
9. All subjects for whom outcome measurements were available received the treatment or control condition as allocated, or where this was not the case, data for at least one key outcome were analyzed by "intention to treat"
10. The results of between-group statistical comparisons are reported for at least one key outcome
11. The study provides both point measurements and measurements of variability for at least one key outcome

Supplementary Figure 1: The methodological quality of assessment for included literature based on the Physiotherapy Evidence Database (PEDro) scale for randomized controlled trails. Note: +, yes; –, no; ?, unclear.
